# Subtle impairments of perceptual-motor function and well-being are detectable among military cadets and college athletes with self-reported history of concussion

**DOI:** 10.3389/fspor.2023.1046572

**Published:** 2023-01-25

**Authors:** Gary B. Wilkerson, Marisa A. Colston, Shellie N. Acocello, Jennifer A. Hogg, Lynette M. Carlson

**Affiliations:** Department of Health and Human Performance, University of Tennessee at Chattanooga, Chattanooga, TN, United States

**Keywords:** mild traumatic brain injury, virtual reality, intra-Individual variability, functional connectivity, executive function, reaction time

## Abstract

**Introduction:**

A lack of obvious long-term effects of concussion on standard clinical measures of behavioral performance capabilities does not preclude the existence of subtle neural processing impairments that appear to be linked to elevated risk for subsequent concussion occurrence, and which may be associated with greater susceptibility to progressive neurodegenerative processes. The purpose of this observational cohort study was to assess virtual reality motor response variability and survey responses as possible indicators of suboptimal brain function among military cadets and college athletes with self-reported history of concussion (HxC).

**Methods:**

The cohort comprised 75 college students (20.7 ± 2.1 years): 39 Reserve Officer Training Corp (ROTC) military cadets (10 female), 16 football players, and 20 wrestlers; HxC self-reported by 20 (29.2 ± 27.1 months prior, range: 3–96). A virtual reality (VR) test involving 40 lunging/reaching responses to horizontally moving dots (filled/congruent: same direction; open/incongruent: opposite direction) was administered, along with the Sport Fitness and Wellness Index (SFWI) survey. VR Dispersion (standard deviation of 12 T-scores for neck, upper extremity, and lower extremity responses to congruent vs. incongruent stimuli originating from central vs. peripheral locations) and SFWI response patterns were the primary outcomes of interest.

**Results:**

Logistic regression modeling of VR Dispersion (range: 1.5–21.8), SFWI (range: 44–100), and an interaction between them provided 81% HxC classification accuracy (Model *χ*^2^[2] = 26.03, *p* < .001; Hosmer & Lemeshow *χ*^2^[8] = 1.86, *p* = .967; Nagelkerke *R*^2 ^= .427; Area Under Curve = .841, 95% CI: .734, .948). Binary modeling that included VR Dispersion ≥3.2 and SFWI ≤86 demonstrated 75% sensitivity and 86% specificity with both factors positive (Odds Ratio = 17.6, 95% CI: 5.0, 62.1).

**Discussion/Conclusion:**

Detection of subtle indicators of altered brain processes that might otherwise remain unrecognized is clearly important for both short-term and long-term clinical management of concussion. Inconsistency among neck, upper extremity, and lower extremity responses to different types of moving visual stimuli, along with survey responses suggesting suboptimal well-being, merit further investigation as possible clinical indicators of persisting effects of concussion that might prove to be modifiable.

## Global view of the concussion problem

Despite an abundance of recent research evidence documenting structural, functional, and neurometabolic abnormalities among collision sport athletes (1), there remains a widespread expectation that most athletes who sustain mild traumatic brain injury (mTBI) will fully recover within a period of 10 days to 2 weeks (2). The term “concussion” specifically refers to mTBI that has been caused by a clearly definable injury event, which produces symptoms that are relied upon for its diagnosis (2). “Head acceleration events” (sometimes designated as repetitive head impacts) that do not produce any readily apparent clinical symptoms have been shown to cause the same types of cumulative pathological changes as those associated with a diagnosed concussion (3, 4). Furthermore, such events appear to have a prolonged effect across the course of a collision sport season that increases susceptibility to concussion (5). Athlete reluctance to report symptoms often precludes diagnosis of concussion, which has been estimated to range from 35% to 62% of athletes who have sustained mTBI (6). Highly sophisticated, expensive, and relatively inaccessible imaging and testing procedures can be used to document alterations in brain pathophysiology, but standard clinical assessment methods have not been found sufficiently sensitive for consistent detection of subtle impairments in cognitive, affective, or motor status (7, 8).

Athletes engaged in collision sports (e.g., football, ice hockey, rugby, soccer) have been found to exhibit changes in a variety of brain health indicators over the course of a season, including evidence of microstructural white matter damage (1, 4, 9–13), impaired functional connectivity (14, 15), biochemical abnormalities (16–18), and elevated levels of specific types of small ribonucleic molecules (19). Very recent research findings strongly suggest that head acceleration events and/or history of concussion can activate a series of complex interactions among regional cerebral blood flow, microRNAs, and dysfunctional metabolic processes that lead to a persistent state of neuroinflammation (20–22). Both the speed and accuracy of brain information processing can be compromised by neuroinflammatory processes that destabilize the transmission of neural signals across white matter tracts and through brain circuits (23, 24). Thus, asymptomatic and apparently healthy athletes who have sustained some level of mTBI from prior concussion(s) and/or head acceleration events may actually possess subtle and prolonged manifestations of a chronic neuroinflammatory response in the brain (20, 25).

## Clinical detection of impaired perceptual-motor function

Primary dependence on vision for guidance of goal-directed behaviors that require motor control makes rapid and accurate processing of neural information essential for effective sport performance and injury avoidance (26, 27). Perception of proprioceptive, vestibular, and auditory inputs to the central nervous system supplement vision in generation of an internal representation of the external environment, which is fundamentally a cognitive process driven by behavioral goals (28). Although perceptual, cognitive, and motor processes can be viewed as somewhat distinct from one another, perceptual-motor efficiency refers to the optimal integration of spatially separated brain processing modules that produce rapid and accurate responses to goal-related stimuli (29). Because otherwise healthy young athletes can recruit additional neural resources to compensate for an impairment of normal processing capability, dual-task assessments are often used to impose combined cognitive and motor demands that approach or exceed the limit of available cognitive reserve (30).

Virtual reality (VR) has been advocated as a means to deliver standardized task stimuli in a manner that can facilitate detection of a subtle and persistent impairment in perceptual-motor function following concussion (31). The earliest documented use of VR for assessment of concussion effects utilized a projection system and force platform to assess postural responses to visual field motion (32–34). Subsequent studies combined the VR projection system with a head-mounted motion tracking system, which demonstrated post-concussion deficits in postural balance, visual-spatial calibration, and reaction time (35–37). “Immersive” VR refers to a system that utilizes a head-mounted display to provide the user with a sense of presence within a three-dimensional environment, which was first used to document subtle deficits in attention and inhibitory control among adolescent athletes who had sustained a concussion within the preceding 2 years (38). Recent research has combined pre- and post-season data derived from an immersive VR system with molecular biology measures relating to energy metabolism and regional cerebral blood flow to document associations of adverse changes with head acceleration events experienced by American college football players (18, 20, 21). A potentially important factor that has not been incorporated into immersive VR research is the effect of physical exertion on cognitive processes, which have been shown to differ from those measured at rest among healthy adults (39). Furthermore, moderate-intensity exercise appears to accentuate cognitive performance differences between otherwise healthy young adults who differ in terms of remote history of concussion (40).

## Interrelated factors characterizing individuals with remote history of concussion

In addition to cognitive dysfunction, altered connectivity among brain networks is believed to play a central role in development of physical, affective, and sleep-related symptoms (41). Behavioral changes associated with prior concussion tend to be subtle and not disruptive to daily function among young athletes, but they can be detected (42). An incremental increase in the severity of symptoms reported after more than one concussion suggests that a vulnerability to future brain injury may exist among athletes with a concussion history (43). Regular baseline screening that includes administration of a symptom inventory with appropriately designed dual-task performance testing has been recommended to potentially identify individuals experiencing long-term adverse effects, which would otherwise remain undetected (44).

Traditional reductionistic study design and frequentist statistical inference for testing specific hypotheses have not been very effective for promoting understanding of complex system phenomena, such as mTBI effects (45). For example, disrupted synchronization of rhythmic neural processes creates variability in behavioral performance that is typically attributed to measurement error or meaningless signal noise (46). Highly heterogenous clinical presentations among individuals with concussion history, non-linear interactions among factors influencing status at a given time point, and the apparent inadequacy of current clinical tests to detect long-term concussion effects further complicate the search for meaningful findings (7, 47). Altered neurological status may be exceedingly difficult to detect when changes are subtle and fluctuations in performance measures are large. Measures with high test-retest reliability will not necessarily yield evidence of dysfunction (48). A sufficiently accurate clinical prediction model for discrimination between normal and impaired neural processing, without requiring expensive and relatively inaccessible brain imaging or neurophysiological testing, could potentially advance the clinical management of mTBI. Thus, the purpose of this exploratory observational study was to identify any VR perceptual-motor performance metric, survey response pattern, or a combination, that would accurately classify cases with self-reported lifetime history of concussion.

## Methods

A total of 75 college students (20.7 ± 2.1 years) from 3 different organizational cohorts voluntarily participated in a virtual reality (VR) test and provided survey responses relating to current well-being (Table 1). The participants included 39 military cadets (29 male, 10 female), 16 college football players, and 20 college wrestlers. The only exclusionary criterion was injury-related restriction from participation in physical activities. History of having sustained at least one concussion at any time in the past was self-reported, along with the number of previous concussions and the estimated number of months since the most recent occurrence of a concussion. All study procedures were approved or confirmed as exempt from review by the Institutional Review Board of the University of Tennessee at Chattanooga.

**Table 1:** Cohort characteristics: count (percentage), mean ± standard deviation, or median (range).

	History of Concussion		No History of Concussion	
Participants	20 (27%)		55 (73%)	
ROTC	6 (15%)		33 (85%)	
Wrestling	5 (25%)		15 (75%)	
Football	9 (56%)		7 (44%)	
Age (years)	20 (18-23)		21 (18-30)	
Sex	Male	Female	Male	Female
	17 (85%)	3 (15%)	48 (87%)	7 (13%)
Height (cm)	181.6 ± 6.3	170.2 ± 5.1	178.0 ± 8.8	162.6 ± 6.9
Body Mass (kg)	91.4 ± 21.9	68.3 ± 17.8	83.1 ± 18.0	62.6 ± 10.5
ADHD (Self-Report)	4 (24%)	0 (0%)	5 (10%)	1 (14%)
Depression (Self-Report)	1 (6%)	1 (33%)	2 (4%)	1 (14%)
Anxiety (Self-Report)	4 (24%)	2 (67%)	6 (13%)	1 (14%)
SFWI^a^ (0-100)	74 (50–100)		92 (44–100)	
ROTC	71 (50–100)		95 (67–100)	
Wrestling	67 (56–86)		86 (44–100)	
Football	75 (58–95)		93 (74–100)	

^a^ Sport Fitness and Wellness Index (Composite of Sport Fitness Index and Overall Wellness Index).

**Figure 1:**
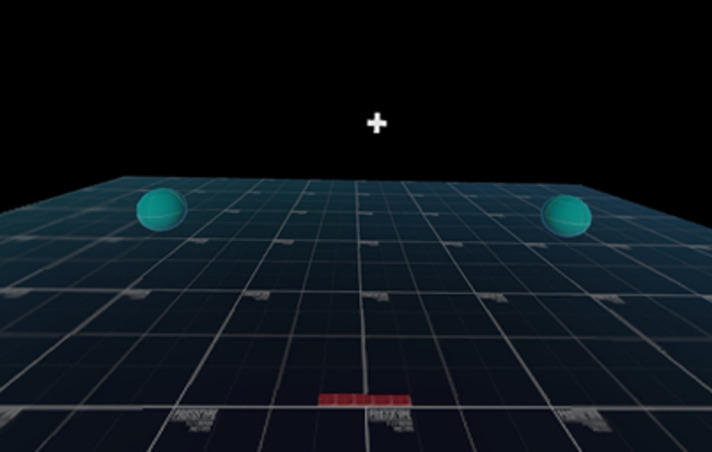
Virtual reality head-mounted display view depicting green response target spheres prior to T-pose calibration procedure. Throughout 40-trial test, response targets located beyond peripheral field of view with head in neutral position (i.e., neck rotation to left or right required to locate correct response target).

The VR test involved 40 reaching/lunging movements in left and right directions in response to the directional motion of horizontally moving white circles that were visible for 500 ms against a black background on a head-mounted display (Pico Neo 3 Pro Eye, Pico Immersive, Ltd., Mountain View, CA). Prior to test initiation, each participant assumed a “T-pose” (i.e., standing upright with horizontally outstretched arms and VR controllers in each hand) to acquire a wingspan measurement that was used to calibrate the reaching/lunging distance required to contact VR response targets (i.e., green spheres; Figure 1). After calibration, the response targets were located 30° anterior to the frontal plane on both sides of the participant's standing position at distances corresponding to 80% of the T-pose measurement, which placed them beyond the peripheral margin of the visual field when looking forward.

**Figure 2:**
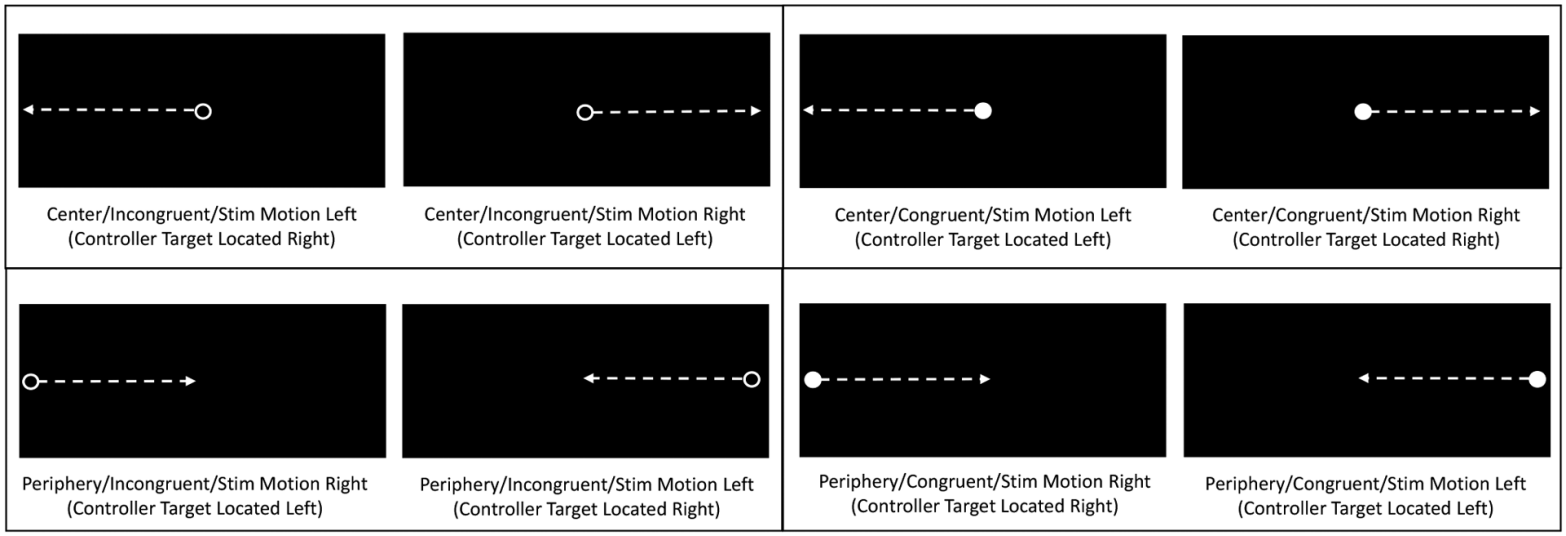
Depictions of head-mounted display views of horizontally moving circles, with arrows representing circle movement directions. Each panel depicts a unique combination of initial moving circle location (center versus periphery) and type (open/incongruent versus solid/congruent). Determination of stimulus type and movement direction required to locate correct response target.

**Figure 3:**
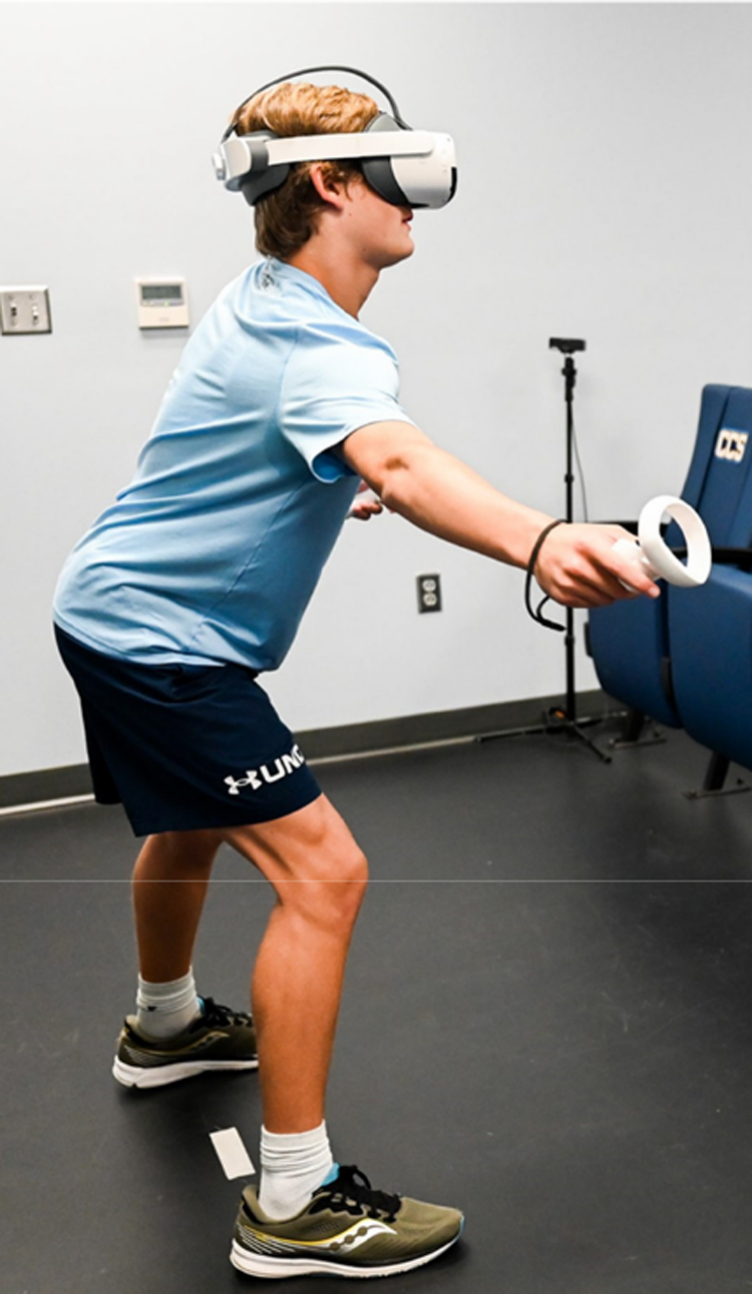
Reaching/lunging to contact virtual reality response target with hand controller.

Stimulus-response instructions were to reach/lunge to hit the green target sphere located in the same direction as the horizontal motion of “solid” white circles (i.e., congruent stimulus-response motion) and to lunge/reach to hit the green target sphere located in the opposite direction to the horizontal motion of “open” circles with a white border (i.e., incongruent stimulus-response motion). Participants were instructed to assume a semi-crouched ready position, with feet positioned at shoulder width and hand controllers against the chest. A central fixation cross appeared on the VR head-mounted display for a duration of 2, 2.5, or 3 s prior to the initiation of each trial to ensure that the eyes were centrally positioned before a moving circle stimulus appeared. On a given trial, a solid or open circle either initially appeared in a central position and moved 60° beyond the left or right peripheral margin of the viewing screen, or it initially emerged from either the left or right peripheral margin and moved 60° to the center of the screen. Thus, 4 types of moving stimuli (i.e., congruent vs. incongruent and central vs. peripheral initial appearance) were randomly presented in rapid succession for total of 40 trials (Figure 2). The speed and direction of each eye, neck, upper extremity, and lower extremity response was derived from an eye tracking camera and an inside-out ultrasonic technology tracking system within the VR head-mounted device, which quantified movements at a 60 Hz sampling frequency. Both an auditory tone and controller vibration were provided as feedback that a response target had been contacted (Figure 3), and which served as a cue to return to the start position for another trial.

Latency was defined as the interval from stimulus appearance to 10 cm of hand controller displacement by the reaching/lunging movement, or the interval from the appearance of one stimulus to the next for trials that did not produce 10 cm of hand controller displacement, which was used to calculate Rate Correct per Second (RCS: number of correct responses divided by the sum of latency values). Response time was defined as the interval from stimulus appearance to maximum displacement (i.e., eye and neck rotation, upper extremity and lower extremity translation), which was used to calculate Conflict Effect (CE) for correct hand controller directional responses (CE = medial of correct incongruent response times minus median of correct congruent response times. Although test-retest reliability has not yet been established for measurements derived from the VR system that we used, performance inconsistency may be an important factor that characterizes suboptimal integration of perceptual-motor processes by the brain. A derived metric, VR Dispersion, was calculated to represent intra-individual variability of standardized (i.e., T-score) values for correct eye, neck, upper extremity, and lower extremity response times for the 4 different types of visual stimuli. An advantage of T-score standardization is representation of standard deviations above and below a standardized mean value of 50 in units of 10. Because prior research has associated “dispersion” of standardized performance values for tasks representing different domains of cognitive function with various neurodegenerative disorders (49–52), the prospectively selected performance metric of primary interest was VR Dispersion.

The Sport Fitness and Wellness Index (SFWI) was administered prior to VR testing to obtain a global estimate of well-being that was derived from a combination of responses to the Sport Fitness Index and the Overall Wellness Index surveys, both of which have been validated as indicators of suboptimal status (40, 53–55). A 0-100 SFWI score was generated from responses to 20 items pertaining to persisting effects of prior musculoskeletal injuries, physical problems, sleep disruption, cognitive limitations, and mood disorders (Supplementary Material). Numerical values ranging from 0 to 5 were assigned to ratings for each of the 20 items, with higher values assigned to descriptors representing greater problem severity/frequency or more recent occurrence (i.e., maximum possible raw score = 5 X 20 = 100). The raw total score was subsequently subtracted from 100 to represent optimal fitness and wellness with a high score, (i.e., low SFWI score corresponding to suboptimal self-rated function and wellness).

### Data analysis

Because this was an observational study, participants were not randomly assigned to groups. To identify any significant differences (*p* < .05) between groups defined by whether or not participants self-reported history of concussion, separate univariate analysis of variance procedures were performed with activity type and sex as categorical variables and height, weight, and age as dependent variables. Because the study was exploratory and the number of available participants was limited, *a priori* estimation of sample size required for detection of a meaningful effect was not performed. Receiver operating characteristic analysis area under curve (AUC) was used to assess the strength of univariable association of potential predictive variables with self-reported history of concussion. Youden's Index was used to identify the optimal cut point for maximum discrimination, which permitted cross-tabulation analysis for determination of sensitivity, specificity, and an odds ratio (OR) and its 95% confidence interval (CI). Backward stepwise logistic regression analysis was used to assess various combinations of continuous predictive variables for maximum discrimination, which included assessment of the possible influences of covariates on the final model. To avoid overfitting, the logistic regression modeling was limited to the strongest 2-factor combination of predictive variables. To explore the potential for a simplified interpretation of the logistic regression result, binary modeling of the strongest 2-factor combination of variables was also performed. Both Pearson r and Spearman *ρ* values were calculated to quantify bivariate correlations between selected performance metrics.

## Results

A history of concussion (HxC) was self-reported by 20 participants (29.2 ± 27.1 months prior, range: 3–96) and 55 participants indicated no history of concussion (NoC). No statistically significant group (HxC or. NoC) by activity type (ROTC, Football, or Wrestling) interaction effect was evident for height (*p* = .809), body mass (*p* = .330), or age (*p* = .970), nor was there a statistically significant by group by sex interaction effect for height (*p* = .985), body mass (*p* = .729), or age (*p* = .798). A history of attention deficit-hyperactivity disorder (ADHD) diagnosis was self-reported by 20% (4/20) with HxC and 11% (6/55) with NoC, which was not a statistically significant difference (Fischer's exact test 2-sided *p* = .420).

**Figure 4:**
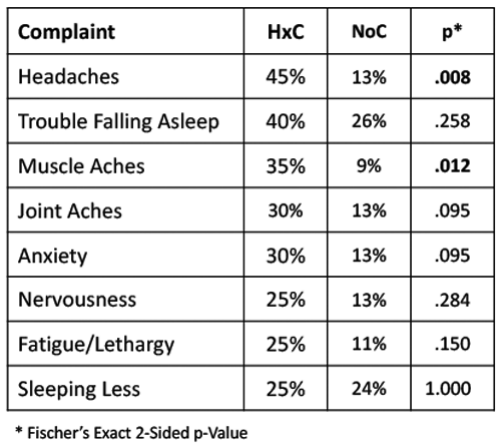
Most common complaints among self-reported history of concussion (HxC) cases, with corresponding frequency of each complaint for no concussion (NoC) cases.

Complete VR test data were acquired for neck, upper extremity, and lower extremity responses, but 11.3% (34/300) of the eye tracking test results for all 4 combinations of visual stimulus types were not captured among 10 participants. The lack of complete eye tracking data may have related to calibration failure. Among the cases with missing eye tracking data, 50% (5/10) were HxC cases. Rather than inserting interpolated values for the missing data, the analysis was limited to potential predictive variables with complete data for the 75 participants. Thus, 12-Metric VR Dispersion was based on the median response times of 3 movement types (i.e., neck, upper extremity, and lower extremity) for each of 4 visual stimulus types (i.e., 12 T-scores). Median and inter-quartile range (IQR) values for 12-Metric Dispersion were 3.5 (2.8, 5.0) for NoC and 4.4 (3.4, 5.6) for HxC. Corresponding values for SFWI score were 92 (56, 57) for NoC and 72 (58, 59) for HxC. Among the 20 cases of self-reported HxC, the most commonly reported factors adversely affecting well-being related to physical discomfort, sleep disruption, and mood disorders are presented, along with corresponding data for the 55 NoC cases (Figure 4). The SFWI musculoskeletal item that provided strongest discrimination documented muscle spasms, stiffness, and/or aching discomfort during activities of daily living (AUC = .692).

**Figure 5:**
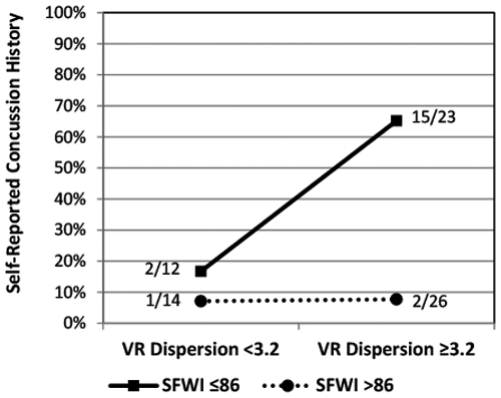
Prevalence of self-reported concussion within subgroups defined by binary categorizations of 12-metric VR dispersion and SFWI.

**Figure 6:**
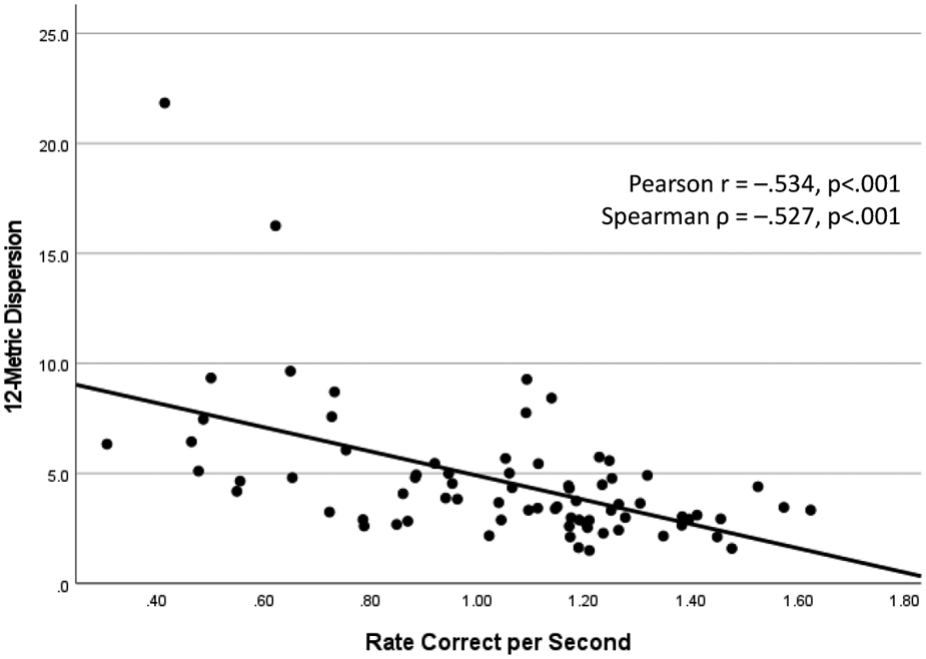
Inverse correlation between 12-metric VR dispersion and rate correct per second.

The SFWI score and each of the VR metrics exhibited a univariable association with HxC (SFWI AUC = .813; RCS AUC = .556; CE AUC = .615; 12-Metric Dispersion AUC = .593). Backward stepwise logistic regression modeling included each VR metric, SFWI, and interaction between each VR metric and SFWI. The best 2-factor model included Dispersion (beta = 2.74, *p* < .001) and the interaction of Dispersion with SFWI (beta = –0.03, *p* < .001), which provided 81% HxC classification accuracy (Model *χ*^2^[2] = 26.03, *p* < .001; Hosmer & Lemeshow *χ*^2^[8] = 1.86, *p* = .967; Nagelkerke *R*^2 ^= .427; AUC = .841, 95% CI: .734, .948). No significant effect was observed for any covariates, including self-reported history of ADHD diagnosis. To assess the accuracy of the 2-factor model across the 3 activity groups, separate receiver operating characteristic analyses of the logistic regression predicted probabilities demonstrated AUC = .813 for military cadets, AUC = .921 for football players, and AUC = .853 for wrestlers. Binary modeling that included 12-Metric Dispersion ≥3.2 (range: 1.5–21.8) and SFWI ≤86 (range: 44–100) demonstrated 75% sensitivity and 86% specificity with both positive (OR = 17.63, 95% CI: 5.00, 62.10), with a clear interaction effect (Figure 5). A potentially meaningful correlation was evident between 12-Metric Dispersion and RCS responses (*r* = –.534, *p* < .001; *ρ *= –.527, *p* < .001; Figure 6). An RCS value ≤0.73 demonstrated only 30% sensitivity, but 89% specificity for discrimination between HxC and NoC (OR = 3.50, 95% CI: 0.98, 12.56). A linear correlation was not apparent between 12-Metric Dispersion and CE (*r* = –.017, *p* = .885; *ρ *= –.119, *p* = .310), but a ≥135 ms binary categorization of CE demonstrated 50% sensitivity and 78% specificity for discrimination between HxC and NoC (OR = 3.58, 95% CI: 1.21, 12.61).

**Figure 7:**
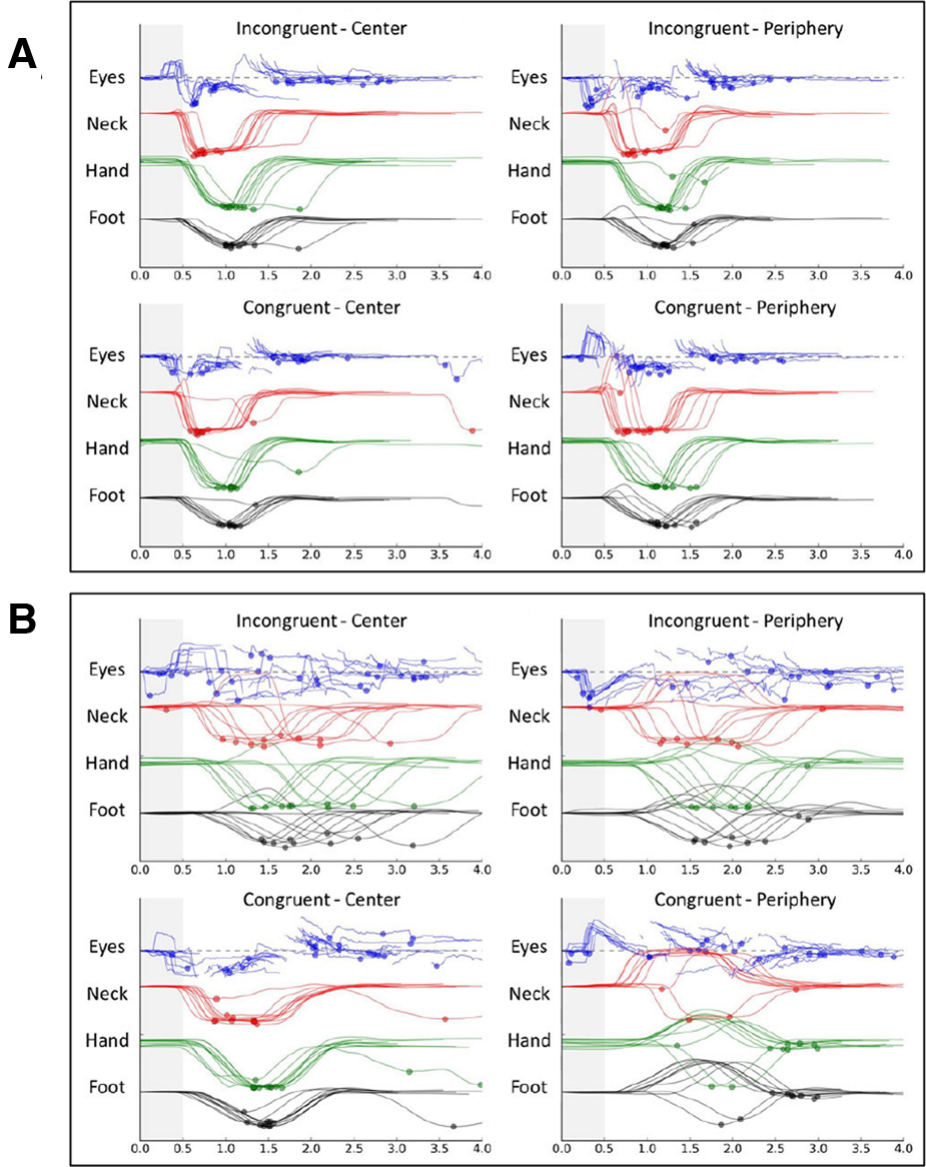
Graphic depiction of within-subject variability in response times for 40-trial virtual reality tests of 2 college football players: low variability (**A**) and high variability (**B**).

To assess VR 16-Metric Dispersion derived from median response times of all 4 movement types (i.e., eye, neck, upper extremity, and lower extremity) and each of 4 visual stimulus types (i.e., 16 T-scores), a logistic regression analysis was completed for the 65 cases with complete eye tracking data (15 HxC and 50 NoC). A 2-factor model that included SFWI (beta = –0.11, *p* < .001) and the interaction of the 16-metric VR Dispersion with SFWI (beta = 0.002, *p* = .069) provided 81.5% HxC classification accuracy (Model *χ*^2^[2] = 18.60, *p* < .001; Hosmer & Lemeshow *χ*^2^[7] = 6.42, *p* = .491; Nagelkerke *R*^2 ^= .377; AUC = .855, 95% CI: .749, .960). Median (and IQR) values for 16-Metric Dispersion were 6.6 (4.8, 8.2) for NoC and 6.4 (5.4, 10.9) for HxC. Median (and IQR) values for 12-Metric Dispersion were 3.6 (2.7, 5.0) for NoC and 4.4 (3.6, 5.6) for HxC, which were nearly identical to the corresponding values for the full cohort of 75 individuals. The same was true for SFWI score median (and IQR) values, which were 92 (57, 60) for NoC and 73 (58, 61) for HxC. To further rule out any important difference between the 10 cases missing eye tracking data from the other 65 cases, Mann-Whitney tests for the strongly skewed distributions failed to identify differences for 12-Metric Dispersion (*p* = .864) or SFWI (*p* = .458). Fischer's exact 2-sided test demonstrated identical proportions of self-reported ADHD (*p* = 1.00). To assess the accuracy of the 2-factor model across the 3 activity groups, separate receiver operating characteristic analyses of the logistic regression predicted probabilities demonstrated AUC = .970 for military cadets, AUC = .730 for football players, and AUC = .800 for wrestlers. Binary modeling of SFWI ≤86 (AUC = .812) and 16-Metric Dispersion ≥10.0 (AUC = .605) did not demonstrate the interaction found for the continuous variables, but ≥1 of the 2 binary factors positive demonstrated 93% sensitivity and 64% specificity (Odds Ratio = 24.9, 95% CI: 3.0, 205.2). A graphic representation of within-subject variability in successive eye, neck, upper extremity, and lower extremity response times is provided for comparison of cases exhibiting low variability and high variability (Figure 7).

## Discussion

Early studies that utilized VR to create a visual illusion of self-motion through a “moving room” projected onto a screen identified an impairment in postural balance among recently concussed individuals, which persisted for at least 30 days after injury (32–34). Because postural sway was not evident in the absence of the visual illusion, nor during testing with the visual illusion that was conducted prior to concussion, the terms “perception-action disintegration” and “visual-motor disintegration” were used to designate the concussion-related impairment (32, 34). Subsequent studies confirmed the effect of visual motion on postural sway among individuals who had sustained a concussion within the preceding 7 to 10 day period (35, 36), as well as an effect on the accuracy and speed of whole-body responses to unpredictable changes VR motion direction (37). Another early study utilized an immersive VR head-mounted display to document impairments in sustained attention, response time, and inhibition of distracting stimuli among adolescents who had sustained a sport-related concussion from 1 to 24 months prior to testing (38). The findings of this study suggest that performance variability in execution of motor responses to different types of visual stimuli displayed within an immersive VR environment represents a behavioral manifestation of inefficient neural processing, which may be a very long-lasting effect of prior concussion.

Several previous studies have documented an association between concussion history and intra-individual variability in the speed of responses requiring discrimination between different types of visual stimuli (62–65), and others have related such behavioral performance inconsistency to neural correlates of inefficient brain function (65–68). Distinctive aspects of this study include the use of moving visual stimuli and the measurement of coordinated eye, neck, upper extremity, and lower extremity responses during whole-body reaching/lunging movements toward VR targets located beyond the peripheral field of view. Moving visual stimuli activate the hV5/MT + motion sensitive area of the brain that is involved in the neural process of generating rapid motor responses (69–71). Horizontally moving circles that impose a cognitive demand (i.e., congruent vs. incongruent stimulus-response instructions) may provide a more perceptually challenging and ecologically valid representation of a real-world dynamic environment than the statically displayed stimuli that are typically used to assess neurocognitive status (72). Given the complexity of the central nervous system and individual variations in its architecture, some amount of within-subject dispersion among different types of stimuli and corresponding responses generated by different body parts is not surprising (73). Thus, previous research that has linked excessive dispersion to early development of aging-related neurodegenerative conditions may have relevance to neural impairment caused by mTBI among adolescents and young adults (46). Consistent with the findings of a recent study involving elite athletes (29), dispersion of multiple standardized values for test metrics representing somewhat different performance domains provided greater discriminatory power for identification of HxC cases than any single test metric. The diffuse and heterogenous nature of mTBI might explain the greater discriminatory power of a multifaceted composite metric compared to a single test result (50).

The interaction between 12-Metric Dispersion and SFWI score for identification of HxC cases depicted in Figure 3 may relate to the manner in which brain networks interact with one another to create integrated circuits (74). The default mode network includes key nodes in the ventromedial prefrontal cortex, posterior cingulate cortex, and precuneus that exhibit a high level of activation in the absence of externally-imposed cognitive processing (75), which is inversely correlated with the activation level of the frontoparietal executive control network during engagement in a goal-directed task (65). The anterior cingulate cortex and insula comprise the salience network, which suppresses the default mode network and activates the executive control network when a goal-relevant external stimulus is detected, and which regulates a number of interactions among distributed nodes of networks that subserve cognitive, sensory, and affective information processing (14). Synchronization of neural signals that oscillate at different frequencies in spatially separated neuron populations provides the mechanism for functional connectivity among brain networks, which is directly related to the relative balance of excitatory and inhibitory activity at neuronal synapses (76–79). Although seemingly paradoxical, performance consistency in goal-directed activities has clearly been linked to global variability in neural signals (80–82). Furthermore, disrupted functional connectivity between the salience network and nodes of other networks may adversely affect perceived well-being related to alterations in autonomic and emotional regulation processes (14, 83, 84).

Numerous studies have demonstrated abnormalities in both the structure and function of the default mode network among individuals who have sustained mTBI (15, 75, 59–85). Microstructural disruption caused by diffuse axon injury can result in hypoconnectivity among network nodes (i.e., reduced within-network connectivity) and/or between networks, but hyperconnectivity (i.e., increased activation of adjacent neuronal pathways) can provide a compensatory mechanism to sustain information processing at the cost of lower metabolic efficiency (15, 61). Patients with chronic mTBI symptoms have been found to have an imbalance in the ratio of cerebral blood flow between nodes of the default mode network (increased perfusion) and those of the executive control network (reduced perfusion), which corresponds to less efficient salience network regulation of functional connectivity between the default mode network and executive control network (86). Because the metabolic cost of sustained hyperconnectivity imposes oxidative stress that can ultimately produce microstructural damage and hypoconnectivity within or between networks (60, 61), the overall effects of different combinations of increased and decreased functional connectivity within brain circuits can change over time. Alteration of normal interactions between the default mode network and salience network following mTBI can disrupt efficient default mode network disengagement and activation of the executive control network, thereby impairing the ability to rapidly detect salient external stimuli (87). Furthermore, inefficient disengagement of the internally focused default mode network may produce elevated awareness of post-concussion symptoms (14). Because the anterior insula component of the salience network integrates visceral, emotional, and autonomic inputs, its interactions with the default mode network and executive control network contribute to the regulation of the heart, the gastrointestinal tract, and emotional responses (84). Microstructural white matter damage may ultimately be related to the findings of a recent report that documented significantly increased risk of medical and behavioral health diagnoses within 5 years of concussion occurrence (88).

Although the 12-Metric Dispersion value for neck, upper extremity, and lower extremity responses provided the best VR indicator of HxC, test results that do not require standardization permit immediate post-test assessment of an individual's performance. Only one prior study has utilized a similar moving circle paradigm for assessment of the ability to cognitively suppress execution of incorrect responses to stimuli that elicit a processing conflict, which demonstrated a lesser Conflict Effect for football players compared to non-athletes (72). Expected associations of HxC with both Conflict Effect and Rate Correct per Second were evident, but lack of a meaningful correlation between Conflict Effect and Rate Correct per Second suggests that they represent the output of different neural processes. The inverse correlation between 12-Metric Dispersion and Rate Correct per Second depicted in Figure 5 suggests that both measures may represent some aspect of the relationship between neural processing and behavioral performance.

The term “neural efficiency” specifically relates quantifiable mental effort (i.e., elevated brain activation) to performance goal attainment, for which the relationship between speed and accuracy of behavioral responses is recognized as a readily attainable estimate (89, 90). The Rate Correct per Second measure offers a numerical representation of performance that adjusts for the inherent speed-accuracy trade-off imposed by choice responses to successive visual stimuli (91). Paradoxically, greater moment-to-moment brain signal variability characterizes a well-functioning neural system that produces accurate, fast, and consistent cognitive performance across multiple performance domains (80, 92). Neural efficiency is closely related to the concept of cognitive flexibility, which refers to the ability to switch between different modes of neural processing in response to changing internal or external environmental demands (81, 82). Conversely, cognitive stability is needed for tasks that require focused attention and inhibition. Metastability refers to an optimal balanced state within the brain that facilitates rapid reconfiguration of networks to enhance information processing capacity (58, 80, 93, 94), which could be expected to produce the inverse relationship between low 12-Metric Dispersion and high Rate Correct per Second that was observed.

Elevated risk for repeated concussion (14, 95), as well as more severe symptoms from repeat concussion (43), suggests that a complex cascade of neural, autonomic, immune, endocrine, metabolic, and epigenetic interactions can have long-term adverse effects without producing symptoms that necessarily raise concern about vulnerability to further brain injury. Such vulnerability may be particularly insidious among collision sport athletes who have experienced default mode network alteration as a result of repeated head acceleration events (15). Consistent with recent work that has documented the predictive value of self-reported HxC with symptom severity among college athletes (42), SFWI responses identified headaches, trouble falling asleep, and anxiety as complaints more commonly reported by individuals with HxC. In contrast to the widely used 22-item Sport Concussion Assessment Tool and the 22-item Post-Concussion Symptom Scale, the Overall Wellness subcomponent of the SFWI includes muscle aches and joint aches as response options that were among the most frequent problems reported by individuals with HxC. The 10-item Sport Fitness subcomponent of the SFWI addresses factors that may impair sport performance and/or elevate risk for musculoskeletal injury. Any response other than “not at all” to a query about “muscle spasms, stiffness, and aching discomfort during activities of daily living” also made a substantial contribution to the observed relationship between SFWI score and HxC. Although musculoskeletal complaints reported by HxC participants may not be causally related to prior concussion, chronic neuroinflammation could certainly play a role in development of heightened symptom awareness (14, 96).

Another aspect of the study findings that warrants consideration is the greater value for 16-Metric Dispersion compared to 12-Metric Dispersion for the subset of 65 participants who had complete eye tracking data. The optimal categorization cut point for 12-Metric Dispersion was ≥3.2 for the full cohort, whereas the cut point for 16-Metric Dispersion for the slightly smaller cohort was ≥10.0. Variability in oculomotor performance has previously been identified as an important indicator of impairment among adults with a history of concussion (97), as well as an association with head acceleration events sustained by high school ice hockey players over the course of a season (57). Decreased oculomotor efficiency from pre- to post-season among athletes participating in collision sports has also been reported (98). Because visual perception is dependent on oculomotor control, regular assessment of the ability to consistency and rapidly detect task-relevant environmental stimuli may be an important consideration for management of injury risk among collision sport athletes.

Surveillance of head acceleration events across a sport season provides an ideal mechanism to study short-term effects, but deployment of wearable microtechnology is an expensive and time-consuming endeavor that is not feasible in most settings. Although reliance on self-reported lifetime history of concussion represents an imperfect means to identify individuals who may possess suboptimal neurologic status, complete medical records are rarely accessible. Given the strong possibility of underreported HxC among NoC participants, the actual predictive value of the VR 12-Metric Dispersion value and SFWI score for identification of individuals with suboptimal neural processing efficiency was more likely underestimated than overestimated. Lack of test-retest reliability data for any of the VR metrics represents an important limitation that needs to be addressed by future work. More research may lead to development of valid and reliable VR metrics that can differentiate individuals with relative resilience to the potentially adverse effects of mTBI from those who possess elevated vulnerability, as well as documentation of the effectiveness of interventions for risk reduction.

## Summary

The findings of this study suggest that inconsistent temporal coordination of motor responses to virtual reality visual stimuli, combined with self-reported physical, affective, or sleep-related problems, can discriminate between military cadets and college athletes who self-report versus deny a lifetime history of at least one concussion. A substantial body of recent evidence suggests that few athletes who have sustained mTBI have fully recovered normal brain function prior to return to sport participation, but current clinical assessment methods lack adequate sensitivity to detect subtle impairments. A state of neuroinflammation that has no readily identifiable signs or symptoms may persist for months or years after mTBI, which makes detection of subtle impairments perceptual-motor efficiency and overall well-being essential for optimal clinical management.

## Data Availability

The raw data supporting the conclusions of this article will be made available by the authors, without undue reservation.
