# Supplementary material for: Subtle impairments of perceptual-motor function and well-being are detectable among military cadets and college athletes with self-reported history of concussion
Source: Front Sports Act Living. 2023 Jan 25;5:1046572. doi: 10.3389/fspor.2023.1046572 (PMC9905443; doi:10.3389/fspor.2023.1046572)
Supplement: Supplementary file 1 [file Datasheet1.pdf]

© 2020

Sport Fitness and Wellness Index

1. Over the past several years, how often have moderate-to-severe muscle and/or joint injuries limited your ability to participate fully in sports-related activities?

Never	Rare	Infrequent	Occasional	Frequent	Persistent
○	○	○	○	○	○

2. Over the past several years, how often has PAIN in any body part limited your OVERALL sport performance capabilities?

Never	Rare	Infrequent	Occasional	Frequent	Persistent
○	○	○	○	○	○

3. To what extent do you feel that previous muscle and/or joint injuries currently limit your speed, power output, and/or endurance?

Not at all	Insignificant	Marginal	Moderate	Substantial	Severe
○	○	○	○	○	○

4. To what extent is your OVERALL ability to perform weightlifting exercises and/or activities that require explosive force output (such as maximum-effort jumping) currently limited by PAIN?

Not at all	Insignificant	Marginal	Moderate	Substantial	Severe
○	○	○	○	○	○

5. To what extent is your ability to perform any SPORT-SPECIFIC SKILL (such as throwing, swinging, or kicking accuracy) currently limited by PAIN?

Not at all	Insignificant	Marginal	Moderate	Substantial	Severe
○	○	○	○	○	○

6. To what extent are you bothered by muscle spasms, stiffness, and/or aching discomfort during routine activities of daily living (such as sleeping, walking, climbing/descending stairs, etc.)?

Not at all	Insignificant	Marginal	Moderate	Substantial	Severe
○	○	○	○	○	○

7. How often do you experience sensations of joint instability, giving-way, and/or sudden pain that create apprehension during rapid and forceful movements (such as pivoting and cutting)?

Never	Rare	Infrequent	Occasional	Frequent	Persistent
○	○	○	○	○	○

8. As a result of participating in sport-related activities, how often do you experience joint aching, limited motion, stiffness, and/or swelling?

Never	Rare	Infrequent	Occasional	Frequent	Persistent
○	○	○	○	○	○

9. To what extent are you bothered by chronic joint symptoms like joint locking, catching, grinding, or persistent aching?

Not at all	Insignificant	Marginal	Moderate	Substantial	Severe
○	○	○	○	○	○

10. Over the past 12 months, to what extent have personal life events created emotional responses (such as sadness, depression, and/or anxiety) that have interfered with your enjoyment of life, ability to concentrate, and/or fulfillment of routine daily responsibilities?

Never	Rare	Infrequent	Occasional	Frequent	Persistent
○	○	○	○	○	○

© 2020

Check (√) each of the problems listed below that have ever interfered with your ability to function in a normal manner during normal day-to-day activities. Also, indicate how recently and how frequently the checked problems within the category have been experienced.

1. **Physical Problems** (check all that apply)

❑ Headaches ❑ Pressure in head ❑ Neck Pain ❑ Muscle aches
❑ Nausea/vomiting ❑ Light sensitivity ❑ Noise sensitivity ❑ Joint aches
❑ Urinary incontinence ❑ Bowel incontinence ❑ General discomfort

Most recent occurrence/frequency of any *Physical Problems* (choose one):

○	○	○	○	○	○
**Current Week Any Extent**	**Past 12 Months Frequently**	**Past 12 Months Infrequently**	**> 1 Year Ago Frequently**	**> 1 Year Ago Infrequently**	**Not at All Over the Past 2 Years**

2. **Sleep/Stamina Problems** (check all that apply)

❑ Sleeping less ❑ Sleeping more ❑ Trouble falling asleep
❑ Fatigue/lethargy ❑ Drowsiness ❑ Feeling slowed down

Most recent occurrence/frequency of any *Sleep/Stamina Problems* (choose one):

○	○	○	○	○	○
**Current Week Any Extent**	**Past 12 Months Frequently**	**Past 12 Months Infrequently**	**> 1 Year Ago Frequently**	**> 1 Year Ago Infrequently**	**Not at All Over the Past 2 Years**

3. **Muscle Control Problems** (check all that apply)

❑ Muscle weakness ❑ Involuntary movements ❑ Muscle twitching ❑ Muscle jerking
❑ Difficulty walking ❑ Tremor (oscillating motions) ❑ Changed handwriting
❑ Trouble using tools ❑ Difficulty using hands or feet ❑ Trouble swallowing

Most recent occurrence/frequency of any *Muscle-Related Problems* (choose one):

○	○	○	○	○	○
**Current Week Any Extent**	**Past 12 Months Frequently**	**Past 12 Months Infrequently**	**> 1 Year Ago Frequently**	**> 1 Year Ago Infrequently**	**Not at All Over the Past 2 Years**

4. **Balance/Orientation Problems** (check all that apply)

❑ Postural swaying/falling ❑ Spinning sensations ❑ Dizziness
❑ Lost in familiar environment ❑ Trouble seeing things properly
❑ Difficulty recognizing faces ❑ Impaired perception of objects

Most recent occurrence/frequency of any *Balance/Orientation Problems* (choose one):

○	○	○	○	○	○
**Current Week Any Extent**	**Past 12 Months Frequently**	**Past 12 Months Infrequently**	**> 1 Year Ago Frequently**	**> 1 Year Ago Infrequently**	**Not at All Over the Past 2 Years**

5. **Abnormal Sensations** (check all that apply)

❑ Vision changes ❑ Tingling ❑ Numbness ❑ Body pains ❑ Other changed sensations

Most recent occurrence/frequency of any *Abnormal Sensations* (choose one):

○	○	○	○	○	○
**Current Week Any Extent**	**Past 12 Months Frequently**	**Past 12 Months Infrequently**	**> 1 Year Ago Frequently**	**> 1 Year Ago Infrequently**	**Not at All Over the Past 2 Years**

© 2020

6. **Mood or Emotional Problems** (check all that apply)

❑ Suppression of emotions ❑ Emotional instability ❑ Depression/sadness
❑ Anxiety ❑ Nervousness ❑ Irritability

Most recent occurrence/frequency of any *Mood or Emotional Problems* (choose one):

◯	◯	◯	◯	◯	◯
**Current Week Any Extent**	**Past 12 Months Frequently**	**Past 12 Months Infrequently**	**> 1 Year Ago Frequently**	**> 1 Year Ago Infrequently**	**Not at All Over the Past 2 Years**

7. **Behavior Control** (check all that apply)

❑ Apathy/lack of motivation ❑ Loss of inhibitions ❑ Intense spirituality ❑ Delusions
❑ Personality changes ❑ Agitation/aggression ❑ Violent outbursts
❑ Obsession/compulsion ❑ Repetitive behaviors ❑ Criminal behavior
❑ Impaired hygiene ❑ Altered eating habits ❑ Hallucinations

Most recent occurrence/frequency of any *Behavior Control Problems* (choose one):

◯	◯	◯	◯	◯	◯
**Current Week Any Extent**	**Past 12 Months Frequently**	**Past 12 Months Infrequently**	**> 1 Year Ago Frequently**	**> 1 Year Ago Infrequently**	**Not at All Over the Past 2 Years**

8. **Memory-Related Problems** (check all that apply)

❑ Misplaced objects ❑ Asking questions repetitively ❑ Missed appointments
❑ Difficulty remembering past events

Most recent occurrence/frequency of any *Memory-Related Problems* (choose one):

◯	◯	◯	◯	◯	◯
**Current Week Any Extent**	**Past 12 Months Frequently**	**Past 12 Months Infrequently**	**> 1 Year Ago Frequently**	**> 1 Year Ago Infrequently**	**Not at All Over the Past 2 Years**

9. **Thinking-Related Problems** (check all that apply)

❑ Planning/organizing difficulty ❑ Multi-tasking difficulty ❑ Problem-solving difficulty
❑ Mental rigidity (inflexibility) ❑ Impulsive responses ❑ Mental Fogginess
❑ Difficulty concentrating ❑ Bad decisions ❑ Confusion

Most recent occurrence/frequency of any *Thinking-Related Problems* (choose one):

◯	◯	◯	◯	◯	◯
**Current Week Any Extent**	**Past 12 Months Frequently**	**Past 12 Months Infrequently**	**> 1 Year Ago Frequently**	**> 1 Year Ago Infrequently**	**Not at All Over the Past 2 Years**

10. **Language-Related Problems** (check all that apply)

❑ Impaired writing ❑ Impaired spelling ❑ Impaired reading
❑ Trouble choosing words ❑ Slurred speech, difficulty articulating words
❑ Stuttering ❑ Incorrect word use/mispronunciation
❑ Increased speech output ❑ Impaired language comprehension
❑ Decreased speech output ❑ Impaired word comprehension

Most recent occurrence/frequency of any *Language-Related Problems* (choose one):

◯	◯	◯	◯	◯	◯
**Current Week Any Extent**	**Past 12 Months Frequently**	**Past 12 Months Infrequently**	**> 1 Year Ago Frequently**	**> 1 Year Ago Infrequently**	**Not at All Over the Past 2 Years**
